# Killing two birds with one stone: A breakthrough in transgene-free gene editing in soybean

**DOI:** 10.1093/plphys/kiae526

**Published:** 2024-10-04

**Authors:** Chong Teng

**Affiliations:** Assistant Features Editor, Plant Physiology, American Society of Plant Biologists; Department of Plant Sciences and Genome Center, University of California, Davis, CA 95616, USA

Gene editing enables precise modifications to the DNA in plants to enhance crop productivity and sustainability. The primary techniques are based on clustered regularly interspaced short palindromic repeats (CRISPR) and CRISPR-associated protein 9 (Cas9). Traditionally, the methods involve stable transformation of CRISPR/*Cas9* containing DNA into target plants via Agrobacterium-mediated transformation (Fig. 1A). However, this method leads to the integration of transgenes including *Cas9* and selection marker genes into plant genomes, raising concerns about off-target effects and regulatory compliance. Marker-assisted gene editing and Transgene-Killer CRISPR can greatly reduce the time and labor in identifying target gene–edited and transgene-free plants (Gao et al. 2016; He et al. 2021), but in many countries, plants transformed by Agrobacterium are classified as genetically modified organisms regardless of whether transgenes are still present. Furthermore, it typically requires dedifferentiation and regeneration of explants, with transformation efficiency being influenced by various factors, including genotypes. As a result, only a limited number of varieties and species have been effectively transformed (Xu et al. 2022).

**Figure 1.**
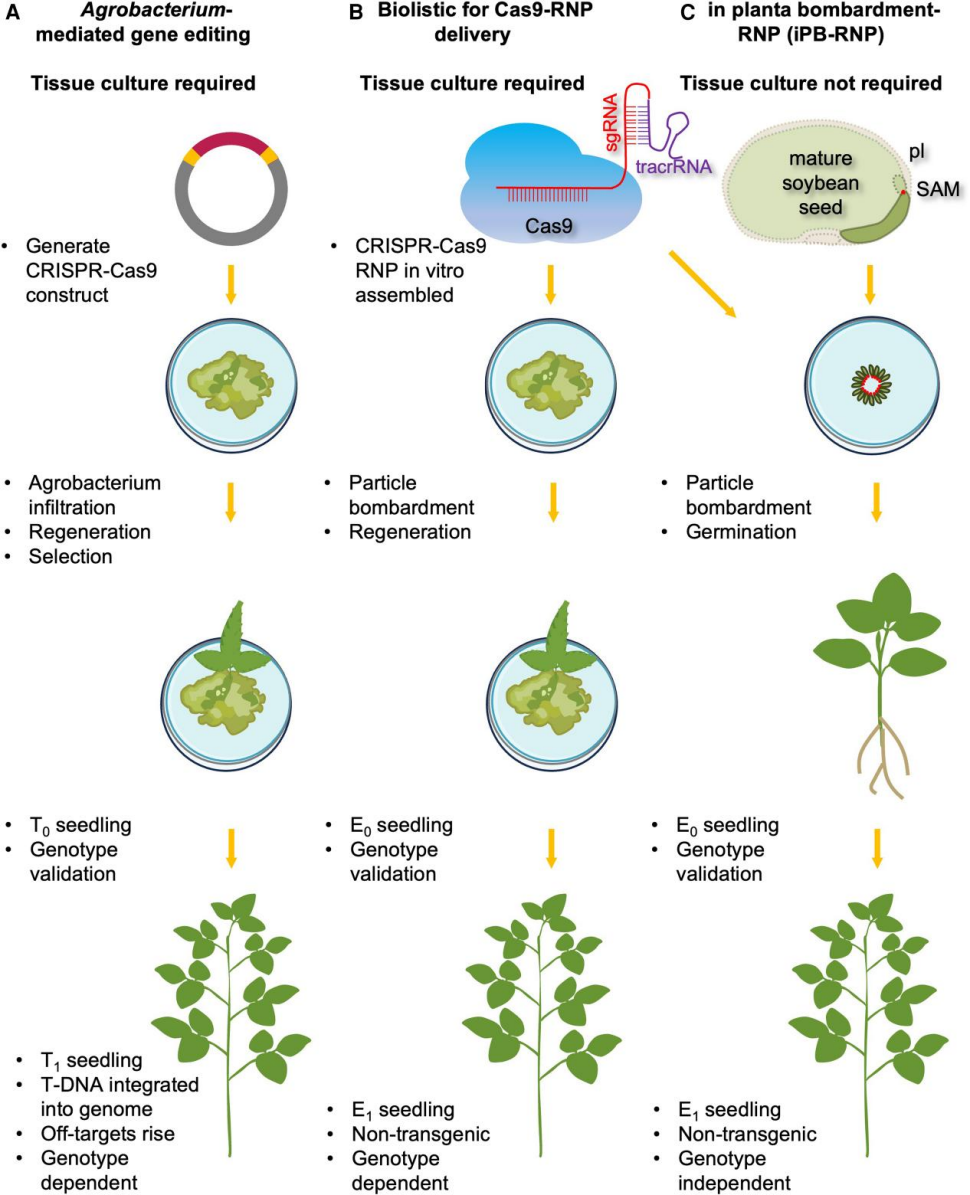
A schematic overview of traditional gene editing methods and iPB-RNP in plants. **A)** Traditional gene editing via stable transformation using Agrobacterium, which requires a binary vector carrying CRISPR-*Cas9* DNA and tissue culture process to regenerate the first generation of transgenic (T_0_) seedlings. Following genotype validation, most of the T_1_ seedlings (75%) exhibit edited gene and integrated CRISPR-*Cas9* DNA in their genomes, which raises concerns about off-target effects. Tissue culture also limits transformation capabilities across most plant species and varieties, restricting this process to only a few crop types. **B)** Delivery of preassembled Cas9-ribonucleoprotein (Cas9-RNP) by particle bombardment into immature embryos or calli in a DNA-free manner. This method likewise requires tissue culture and is applicable only to specific crop types. **C)** iPB-RNP delivers Cas9-RNP via particle bombardment directly into the stem cells of the SAM of mature soybean embryos. Embryonic axes (solid outline) are isolated with the plumule (pl) removed to expose SAM (red dots). Sixteen embryonic axes are targeted in one bombardment, then germinate and grow directly into the first generation of edited seedlings (E_0_). Following genotype screening and validation, the transgene-free E_1_ seedlings are confirmed mutants. This method is likely applicable in a larger collection of varieties and species.

An alternative approach involves preassembling *Cas9* and guide RNAs into ribonucleoprotein (RNP) complexes in vitro. The assembled RNP particles are delivered into plant cells via particle bombardment, producing organisms containing no foreign DNA, which are considered nontransgenic. The RNP method was first utilized in bacteria and mammalian cells as a strategy to detect and treat human diseases (Gasiunas et al. 2012; DeWitt et al. 2017; Li et al. 2023). In plants, several studies have demonstrated successful delivery of Cas9-RNP via bombardment, followed by the regeneration of candidate mutants through tissue culture (Gu et al. 2021). However, this method is currently limited to a few crop types or varieties (Fig. 1B). Recently, successful direct delivery of Cas9/guide RNA RNP into mature wheat embryos via bombardment was reported (Kumagai et al. 2021), but its feasibility in other species, such as dicots, remains unexplored.

In a recent issue of *Plant Physiology*, Kuwabara et al. (2024) succeeded in knocking out an allergenic gene in soybean by in planta bombardment (iPB-RNP). Kuwabara et al. isolated mature embryos from germinating soybean seeds as explants for bombardment targeting the shoot apical meristem (SAM). Remarkably, the mature embryos that underwent RNP bombardment did not require regeneration and were able to germinate and develop directly into the first generation of genome-edited (E_0_) plants (Fig. 1C). Among 1,100 E_0_ plants analyzed, edits in the target gene were detected in the leaves of 66 plants (approximately 6%). Some of these E_0_ plants exhibited chimeric or bi-allelic mutations, indicating that mutagenesis can occur simultaneously in the same stem cell. More importantly, the genotypes of the second generation of genome-edited (E_1_) plants largely matched those of the E_0_ plants, with no instances of chimeric mutants observed in E_1_ plants. Kuwabara et al. applied this optimized method to 4 additional soybean varieties. Interestingly, the overall efficiency of iPB-RNP (0.4% to 4.6%) is comparable with that reported in wheat (Kumagai et al. 2021), suggesting that iPB-RNP is promising for applications in various other cultivars and potentially in different species.

In summary, iPB-RNP enables target-specific mutagenesis in soybean, a dicot species, in a transgene-free and regeneration-independent manner by delivery of Cas9-RNP directly into the stem cells of the mature embryonic axes. This innovative method could pave a way for broader applications in plants, such as commercialization of gene-edited soybeans and facilitating gene editing across diverse plant species. Additionally, it may improve technologies such as targeted knock-in in plants, which requires high efficiency of simultaneous mutagenesis. Ultimately, the advancements offered by iPB-RNP could significantly enhance future research efforts and contribute to the sustainability of agriculture.

## Data Availability

No new data were generated or analysed in support of this research.
